# AAPM Medical Physics Practice Guideline 8.a.: Linear accelerator performance tests

**DOI:** 10.1002/acm2.12080

**Published:** 2017-05-26

**Authors:** Koren Smith, Peter Balter, John Duhon, Gerald A. White, David L. Vassy, Robin A. Miller, Christopher F. Serago, Lynne A. Fairobent

**Affiliations:** ^1^ Mary Bird Perkins Cancer Center Baton Rouge LA USA; ^2^ MD Anderson Cancer Center Houston TX USA; ^3^ e+ OncoLogics Lafayette LA USA; ^4^ Colorado Associates in Medical Physics Colorado Springs CO USA; ^5^ Spartanburg Regional Healthcare System Spartanburg SC USA; ^6^ Northwest Medical Physics Center Lynnwood WA USA; ^7^ Mayo Clinic Jacksonville FL USA; ^8^ AAPM Headquarters Staff Alexandria VA USA

**Keywords:** quality assurance

## Abstract

**Purpose:**

The purpose of this guideline is to provide a list of critical performance tests in order to assist the Qualified Medical Physicist (QMP) in establishing and maintaining a safe and effective quality assurance (QA) program. The performance tests on a linear accelerator (linac) should be selected to fit the clinical patterns of use of the accelerator and care should be given to perform tests which are relevant to detecting errors related to the specific use of the accelerator.

**Methods:**

A risk assessment was performed on tests from current task group reports on linac QA to highlight those tests that are most effective at maintaining safety and quality for the patient. Recommendations are made on the acquisition of reference or baseline data, the establishment of machine isocenter on a routine basis, basing performance tests on clinical use of the linac, working with vendors to establish QA tests and performing tests after maintenance.

**Results:**

The recommended tests proposed in this guideline were chosen based on the results from the risk analysis and the consensus of the guideline's committee. The tests are grouped together by class of test (e.g., dosimetry, mechanical, etc.) and clinical parameter tested. Implementation notes are included for each test so that the QMP can understand the overall goal of each test.

**Conclusion:**

This guideline will assist the QMP in developing a comprehensive QA program for linacs in the external beam radiation therapy setting. The committee sought to prioritize tests by their implication on quality and patient safety. The QMP is ultimately responsible for implementing appropriate tests. In the spirit of the report from American Association of Physicists in Medicine Task Group 100, individual institutions are encouraged to analyze the risks involved in their own clinical practice and determine which performance tests are relevant in their own radiotherapy clinics.

The American Association of Physicists in Medicine (AAPM) is a nonprofit professional society whose primary purposes are to advance the science, education, and professional practice of medical physics. The AAPM has more than 8000 members and is the principal organization of medical physicists in the United States.
The AAPM will periodically define new practice guidelines for medical physics practice to help advance the science of medical physics and to improve the quality of service to patients throughout the United States. Existing medical physics practice guidelines will be reviewed for revision or renewal, as appropriate, on their fifth anniversary or sooner.
Each medical physics practice guideline represents a policy statement by the AAPM, has undergone a thorough consensus process in which it has been subjected to extensive review, and requires the approval of the Professional Council. The medical physics practice guidelines recognize that the safe and effective use of diagnostic and therapeutic radiology requires specific training, skills, and techniques, as described in each document. Reproduction or modification of the published practice guidelines and technical standards by those entities not providing these services is not authorized.
The following terms are used in the AAPM practice guidelines:

- Must and Must Not: used to indicate that adherence to the recommendation is considered necessary to conform to this practice guideline.
- Should and Should Not: used to indicate a prudent practice to which exceptions may occasionally be made in appropriate circumstances.

Approved January 19, 2017

## 1 INTRODUCTION

A comprehensive quality management program in a radiotherapy clinic utilizing external beam radiation therapy will include performance testing of a linear accelerator (linac). The linac must be tested routinely to ensure that current performance parameters have not deviated from baseline clinical parameters acquired at the time of acceptance of the machine. More importantly, it must be validated that the beam models in the treatment planning system (TPS) are still appropriate for the linac in its current operating state.

The technology and control systems within a linac are rapidly evolving and new features emerge frequently to assist the user in accurately and efficiently treating patients. The specific choice and use of technology on a linac will depend on the types of diseases treated, the clinical workload, and workflow. The performance tests on a linac should be selected to fit the clinical patterns of use of the accelerator and care should be given to perform tests which are relevant to detecting errors related to the specific use of the accelerator.

## 2 GOALS AND RATIONALE

This document is part of a series of medical physics practice guidelines commissioned by the American Association of Physicists in Medicine (AAPM) intended to describe acceptable standards for various aspects of clinical medical physics. The implementation of comprehensive quality assurance (QA) programs recommended in AAPM Task Group Reports^1^, ^2^, ^3^ is encouraged. The purpose of this guideline is to provide a list of critical performance tests in order to assist the Qualified Medical Physicist (QMP) in establishing and maintaining a safe and effective QA program that matches the clinical use of the accelerator. The QMP is responsible for choosing and implementing appropriate tests.

Committee members of this guideline reviewed the current protocols for performance tests on a linac. A risk assessment was performed on currently recommended tests in order to identify those tests which will enable the greatest detection of errors, the delivery of high‐quality radiation therapy and reflect the characteristics of modern technology.

This report describes dosimetry, mechanical, and safety tests for C‐arm type linacs only. Specialized systems such as CyberKnife^®^ or TomoTherapy^®^ are not considered here. The scope of this guideline does not include tests for on‐board imaging equipment. Imaging tests are essential in a linac QA program and they are addressed in previous reports.^3^, ^4^, ^5^

Implementation notes are included for each recommended test so that the QMP can understand the overall goal of each test. However, this guideline is not intended to be a “how to” document. Suggestions will be made on what types of devices are helpful and suitable for measurement, but the choice of measurement equipment and technique is ultimately the responsibility of the QMP.

## 3 INTENDED USERS

The intended users of this report are QMPs who are conducting linac performance tests or those that are designing a QA program for linacs and seek to understand the critical tests needed to detect errors and ensure safe and high quality external beam radiation therapy delivery.

Administrators, manufacturers of linacs, personnel representing accrediting bodies and state regulators are also encouraged to use this guideline as a reference in understanding an institution's use of equipment and necessary tests chosen by the QMP to maintain the equipment.

## 4 STAFF QUALIFICATIONS AND RESPONSIBILITIES

A Qualified Medical Physicist is defined by AAPM Professional Policy 1.^6^ The QMP shall be able to independently perform all of the required duties in the field of therapeutic medical physics, including designing and maintaining an overall QA program.

The QMP must design and direct all QA activities, aid in the performance of tests or analysis if needed and assume professional responsibility for the work done.^7^ The QMP may delegate certain QA responsibilities to qualified personnel. The QMP is responsible for reviewing and promulgating the outcome of tests and ensuring that the results are meeting set tolerances.

## 5 DEFINITIONS AND ABBREVIATIONS

### 5.A Abbreviations

3D Conformal, three‐dimensional conformal radiation therapy.

AAPM, American Association of Physicists in Medicine.

CAX, central axis.

CBCT, cone‐beam computed tomography.

D, (lack of detectability) probability of not detecting a failure; as used in FMEA analysis.

DICOM, digital imaging and communications in medicine.

DOFs, degrees of freedom.

EPID, electronic portal imaging device.

FMEA, failure mode and effects analysis.

IEC, International Electrotechnical Commission.

IGRT, image guided radiation therapy.

IMRT, intensity modulated radiation therapy.

MLC, multi‐leaf collimator.

MU, monitor unit.

O, (occurrence) frequency of failure; as used in FMEA analysis.

OAF, off‐axis factor.

ODI, optical distance indicator.

PDD, percent depth dose.

QA, quality assurance.

QMP, qualified medical physicist.

RPN, risk priority number.

S, (severity) the severity of a failure; as used in FMEA analysis.

SBRT, stereotactic body radiation therapy.

SRS, stereotactic radiosurgery.

SSD, source‐to‐surface distance.

TBI, total body irradiation.

TPR, tissue phantom ratio.

TPS, treatment planning system.

TSET, total skin electron therapy.

VMAT, volumetric modulated arc therapy.

## 6 PERFORMANCE TEST REVIEW

### 6.A FMEA methodology

A risk assessment was performed on tests from current task group reports on linac QA following the failure mode and effects analysis (FMEA) approach.^8^, ^9^, ^10^ Reviewed tests were primarily from the report of AAPM Task Group 142, “Task Group 142 report: Quality assurance of medical accelerators”.^3^ The goal of the risk‐based analysis was to highlight those tests on a linac that are most effective at maintaining safety and quality for the patient per the report of AAPM Task Group 100 “Application of Risk Analysis Methods to Radiation Therapy Quality Management”.^8^ Each test (or each clinical parameter being tested) was considered a potential failure mode on a linac and was scored for Occurrence (O), Severity (S), and lack of Detectability (D) of a failure.

Each committee member submitted risk assessments scores for O, S, and D. Each committee member also engaged colleagues, such that a total of 25 practicing medical physicists participated in the risk assessment as scoring participants. The range of years of experience among the scoring participants was 5–37 yr with a median of 20 yr. The scoring participants also have experience in different types of institutions: university/academic, private/community hospital, government, and medical physics consulting groups from different parts of the country. In doing so, the scoring represents the perspective from various patient populations, technologies, age of equipment, types of treatments (i.e., 3D conformal, IMRT, SRS), and diversity of treatments. A scoring table was derived from published tables in order to have a common understanding of the definition and range of O, S, and D.^8^, ^9^

Scoring participants assigned occurrence scores to performance tests using their experience of failure rates for the clinical parameter in question. For example, scoring participants considered how often the optical distance indicator (ODI) test has fallen out of tolerance in their experience.

Scoring participants assigned a severity score to each performance test. In order to assign a severity score, scoring participants assumed that the clinical parameter in question was not being tested at the recommended frequency and was out of tolerance. We then considered the severity of harm to a patient if the patient were treated with an out of tolerance clinical parameter (e.g., the ODI is off by greater than the tolerance value and therefore the patient's source‐to‐surface distance (SSD) could be off by the same amount). To score severity, we made assumptions about how far clinical parameters are out of tolerance when they do fail. For example, committee members reported that the ODI is typically out of tolerance by a few millimeters and not as much as a few centimeters.

For detectability, scoring participants used their knowledge of other tests being performed or knowledge of interlocks/alarms to decide if a clinical parameter failure could be detected via another pathway (besides the performance test itself). For example, scoring participants considered how likely it is to detect that the ODI is out of tolerance if this parameter were not tested daily.

We determined the average score for O, S, and D from each scoring participant and used this to determine an average risk priority number (RPN) value (*RPN* = *O*·*S*·*D*) for each performance test that was scored.

### 6.B Risk assessment scores

The average RPN scores from 25 scoring participants are presented in Appendix I. The scores are sorted by test frequency and highest RPN score. The RPN scores were also normalized by the highest score for a particular testing frequency (e.g., daily, weekly, etc.) and are presented as relative RPN scores.

Table 1 shows the practice guideline's ranking of daily and monthly TG142 tests compared to O'Daniel's FMEA Analysis of TG142.^11^ The method of scoring between the two works is different; O'Daniel chose not to include detectability stating that if the test was not performed, the assumption is that the failure cannot be detected. To determine occurrence, actual data from three linacs over a period of 3 years were analyzed yielding a minimum detectable occurrence rate of 0.04%. Severity rankings were determined by modeling errors in the treatment planning system.

**Table 1:** Comparative risk analysis of TG142

MPPG 8.a.^a^		O'Daniel^b^	
RPN	Performance tests	RPN	Performance tests
**Daily tests scored in both works**			
132	Output constancy	180	Output constancy
83	Laser localization	140	Laser localization
70	Collimator size indicator	60	Distance indicator (ODI) @iso
41	Distance indicator (ODI) @iso	40	Collimator size indicator
**Monthly tests scored in both works**			
143	Output constancy	180	Output constancy
86	Laser localization	140	Laser localization
73, 66	Light/rad field coincidence (asym, sym)	100	Light/rad field coincidence
72, 67	Jaw position indicators (asym, sym)	60	Distance check device
61	Distance check device	40	Jaw position indicators
55	Treatment couch position indicators	40	Treatment couch position indicators

a Medical Physics Practice Guideline 8.a.

b O'Daniel, AAPM Spring Clinical Meeting 2015.^11^

The RPN scores are presented from each work for comparison. For commonly scored tests, the rank order of daily and monthly tests are similar between this work and O'Daniel's results. The highest ranking tests were the same in both works for daily and monthly performance tests (output constancy, laser localization). Differences in ranking order exist in the mid‐level and lower ranking tests.

### 6.C Relative risk compared to other clinical processes

Failures in hardware and software systems on a linac can happen and the QMP must design a QA program that includes tests designed to detect failures. However, hardware and software system functions on a linac represent just one portion of the extensive process map that comprises the external beam treatment paradigm.^10^ The relative risks of hardware and software errors are lower than risks due to human process‐related errors, lack of standardized procedures, and inadequate training of staff.^12^ While we must be diligent to ensure that risks of hardware and software errors are kept low and minimally contribute to the overall goal of delivering dose to the target with a high degree of accuracy,^13^, ^14^ the linac performance testing portion of our QA programs should be efficient so that time and resources can be dedicated to other areas where FMEA indicates errors with a higher score can also occur.

## 7 MINIMUM REQUIRED RESOURCES AND EQUIPMENT

The authors do not recommend a specific tool or technique to perform each test; rather, we provide guidance on methods to achieve the goal of the test. The test procedure and equipment utilized must be capable of both accurate measurements as well as measuring to the level of the stated criteria or test tolerance. It is assumed that the most basic tools are available to the QMP.

There exists a wide variety of equipment and software tools to aid the QMP in performing, analyzing, and interpreting measurements accurately and efficiently. They can be costly, but actually represent a small percentage of the revenue generated by a single linear accelerator over its lifetime. The budget for a new linac and annual operating budgets should include the cost of such measurement equipment and software.

Administrators and department managers should understand the cost‐benefit of purchasing these tools and the time savings that they provide the QMP. It has been shown that some quality control measures are more effective than others^15^ and the QMP should allocate the appropriate amount of time on testing that is relative to the risks involved.

## 8 DEVELOPING A QUALITY MANAGEMENT PROGRAM FOR PERFORMANCE TESTS

### 8.A Reference/baseline data

Detection of most linac performance problems requires comparison to some baseline, or reference, dataset. This reference dataset should be chosen carefully. Clinical treatment decisions are primarily based on dose modeling done in a TPS thus, it is reasonable to compare ongoing linac performance to commissioned TPS data (i.e., TPS data become the reference data). In doing so, the link between what the physician approves to be delivered, and what the linac actually delivers, is validated. In addition, clinically relevant tolerances may be used as opposed to best achievable tolerances.

That being said, an alternative approach is to compare measurement results to data collected at the time of commissioning, as TG142 and the report of AAPM Task Group 106 suggest.^3^, ^16^ Then, the beam data collected at the time of commissioning is used as the reference data. If the QMP chooses the latter approach, it is their responsibility to ensure that the commissioning data agree with the TPS model on an annual basis, as recommended in TG106. This extra step is required in the latter approach so that there is always a link between routinely measured and TPS data. Regardless of the approach chosen, the overall goal is to ensure that during clinical usage the delivered and calculated doses agree within 5% including the uncertainty associated with absolute calibration.

A water tank is typically used for beam measurements at commissioning and annual testing. For more routine measurements, such as profile constancy on a daily or monthly basis, it is easier to use a device other than a water tank. For example, a secondary measurement system may be used for monthly measurements and a tertiary system may be used for daily measurements. In this case, it is necessary to create a reference dataset that has been appropriately verified by TPS data or compared to an absolute standard. An effective approach for creating a routine reference dataset (or creating a baseline) is outlined in the process below:

- Perform annual beam measurements
- Compare results of annual measurements to TPS data, commissioning data that are verified by TPS data, or absolute standards (TG51 calibration standards)
- Ensure results are within acceptable tolerance and resolve differences (if any)
- Once annual beam measurements are verified, make measurements with the routine device/method (secondary and tertiary measurement systems). Ideally, this occurs in the same measurement session on the same day. The data acquired from this measurement are now the reference dataset that effectively becomes the baseline for comparison for routine measurements.

It is the responsibility of the QMP to ensure that all reference datasets are appropriately used and verified against absolute standards (i.e., the TPS) on at least an annual basis.

### 8.B Isocenter

One critical piece of reference data that is not in the TPS is the location of the radiation isocenter. The location of isocenter of the accelerator, both the mechanical and radiation isocenter and the congruence of the two points, are defined and established at the time before commissioning measurements commence. Dosimetric parameters critical to defining the model for the accelerator in the TPS will depend on having accurate knowledge of the isocenter position. The QMP should decide on the appropriate method to establish the isocenter position at the time of acquiring commissioning data. The QMP should then decide on an appropriate reference frame to “find” isocenter on a routine basis and this should be the original reference frame or be tied to the original reference frame decided upon at the time of acceptance/commissioning.

The reference frame for routine tests may be the lasers for some institutions/machines or it may be an external device that is attached to the accelerator. Regardless of method or device used, the QMP should have full knowledge of the reference frame that is used to establish baseline values with which to compare when testing other clinical mechanical parameters. The first step in measuring clinical parameters related to the mechanical accuracy of the accelerator is to ensure that the reference frame still accurately defines the isocenter position to within the desired tolerance. For example, if the lasers are used to reference the isocenter, the lasers should be tested against the radiation/mechanical isocenter before any other mechanical tests are performed. It is the QMP's responsibility to ensure that any adjustments made to mechanical parameters are appropriate and tied to the reference frame used for baseline and routine measurements. This requires excellent communication and documentation about the appropriate conditions for performing mechanical testing on each accelerator. It is recommended that the QMP establish a common method for all accelerators within an institution in order to avoid confusion especially when responsibility for routine testing may be shared or passed on to different personnel.

### 8.C Performance tests based on clinical practice

A robust QA program will be based on the individual needs of a clinical practice. An all‐encompassing table does not exist to dictate the entirety of performance tests that should be performed on a linac to ensure the most accurate and safe treatment for all patient types and all delivery techniques. This guideline provides a list of critical tests that should be considered. However, clinical practice and use of the technology can vary widely on each linac. The QMP is ultimately responsible for deciding which tests are prudent to perform based on their clinical practice. The report from AAPM Task Group 100^8^ provides excellent tools to assist the QMP with this task.

Image guidance techniques used in conjunction with C‐arm linacs have become prevalent in clinical practice to accurately align the patient for treatment. Imaging tests that are closely tied to the mechanical aspects of a linear accelerator (i.e., imaging isocenter vs treatment isocenter) are critically important. The reader is referred to previous reports^3^, ^4^, ^5^ for suggested performance tests for imaging equipment to ensure accurate alignment of the patient as well as the coincidence of the imaging and treatment isocenters. In a clinical practice where the use of imaging to align the patient is emphasized and used more frequently than other alignment techniques such as the lasers or the ODI, the user may consider decreasing the frequency of testing the lasers or the ODI. In this setting, QA of the imaging equipment becomes more critical and should be emphasized on a frequent testing basis.

It is also common to use C‐arm linacs to treat patients with a stereotactic radiosurgery (SRS) or a hypofractionated [stereotactic body radiation therapy (SBRT)] treatment regimen.^17^, ^18^ In this clinical setting, the QMP should refer to protocols specifically designed for performance tests in a stereotactic setting in order to achieve a higher degree of accuracy than that needed for regularly fractionated patients.^19^, ^20^ In addition, the QMP may choose to do additional testing (i.e., Winston‐Lutz test) on the day of the treatment for stereotactic/hypofractionated treatments to ensure that the mechanical alignment of the radiation isocenter is appropriate for such patients.

### 8.D. Vendor provided tests and tools

Many equipment vendors provide recommended QA and safety tests for their equipment. These tests may be a useful reference for the QMP, who should be familiar with the recommendations. However, it is the responsibility of the QMP to evaluate which of these tests are appropriate for their institution and the usage of each machine. Also, vendor tests are rarely a complete, comprehensive set of tests. For example, they often do not include safety tests in clinical context, such as door interlock checks. Thus, it is incumbent on the QMP to supplement vendor supplied tests when they do not span all needed characteristics of the machine QA program. In a multivendor environment, the QMP may choose to use a common test across multiple vendor machines rather than perform each of the vendors’ recommended tests. This is easier on the technical staff as they do not need to learn different tests for each machine as well as providing a common QA base across all machines.

It is recommended that each vendor provide recommended performance and safety tests that cover any aspect of their systems that may be unique. This will assist the QMP in developing their clinical practice and an equipment‐specific QA program. This also takes advantage of vendor's unique understanding of their machine and its operation. Just as QMPs should not rely on the vendor for establishment of the QA program, the vendor should not rely on the QMP to develop all test procedures with no vendor‐specific guidance. The vendor and the QMP should work together in the development of effective and efficient QA programs for each institution.

Vendor tests are often based on vendor supplied tools. Some of these tools are unique to that particular machine and are required. Other tools are generic commercially available tools supplied by the vendor as part of the purchase of the machine and tied to the vendors’ service procedures. It is the responsibility of the QMP to decide if these tools are appropriate beyond the vendors’ service procedures. The QMP may choose to use tools different from the vendor for acceptance testing and/or routine testing. This may be done to standardize equipment across different vendors or to choose equipment that provides more data and/or is easier to use than the vendor supplied device.

### 8.E Performance tests after maintenance

There are some tests that should be performed after general or specific maintenance on an accelerator to ensure that clinical parameters have not changed either intentionally or inadvertently. The QMP should decide which tests are appropriate depending on the type of work done and the potential for a change in performance. The QMP is expected to have a working knowledge of their linac and its sub‐systems so as to make reasonable decisions on what tests need to be done after each type of repair. The service engineers are a valuable resource to help in understanding how the work done may affect clinical parameters. The QMP should have full knowledge of any and all service work being performed on an accelerator and to have a working system in place for the notification, to the physicist, of completion of work and approval by the physicist before the linac returns to service.

## 9 LINAC PERFORMANCE TESTS

### 9.A QMP review of all tests

In addition to designing the performance testing program, the QMP must have working knowledge sufficient to know how test results and beam parameters may be interrelated. For example, if the daily output were to fail, the root cause may be due to a change in the beam energy and not a drift in the monitor chamber. The QMP should also understand the linac's ability and limitations in self‐detecting errors.

Several performance tests are recommended at different frequencies (i.e., daily, monthly, and annually) and are performed by different personnel perhaps using different equipment. The QMP should ensure that all tests being performed for a clinical parameter are considered before making any adjustments and potentially changing any baseline values. When finding that a clinical performance parameter is out of tolerance and needs to be adjusted, it may be necessary to go back several steps in the QA process to ensure that adjusting this parameter did not have any effects on any other clinical parameters. The QMP should be especially mindful of how any adjustments affect the agreement between the machine performance and the TPS calculations.

### 9.B Recommended tests

Recommended tests are described below in Tables 2, 3, 4, 5, 6. The tests were chosen based on the results from the risk analysis and the consensus of this committee. In some cases, the committee chose to include a lower ranking test or to exclude a high ranking test based on clinical reasons and the experience of the committee members. For most tests, our recommendations are consistent with the risk assessment results. We ultimately advise the QMP to implement tests that are relevant to their clinical practice.

**Table 2:** Dosimetry Tests

Item	Test	Frequency	Tolerance
D1	Photon and electron output constancy	Daily^a^	3% of baseline
		Monthly	2% of baseline
		Annual	1% of TG51
D2	Photon and electron beam profile constancy	Daily^a^	2%
		Monthly	2%
		Annual	2% of TPS OAFs^b^
D3	Electron beam energy	Monthly	2 mm
		Annual	2 mm
D4	Photon beam energy	Monthly	1% of PDD/TPR (relative change in value)
		Annual	1% of PDD/TPR at reference depth
D5	Dynamic delivery control	Monthly	3% of open field dose
D6	Photon MU linearity (output constancy)	Annual	2% >10 MU for open field; 2% for segmented field
D7	Electron MU linearity (output constancy)	Annual	2% for clinical range
D8	Photon output vs dose rate	Annual	2%
D9	Photon and electron output vs gantry angle	Annual	2% of IEC gantry 0° output
D10	Photon and electron OAF vs gantry angle	Annual	2% of OAFs at IEC gantry 0°
D11	Arc mode (expected MU, degree)	Annual	2% of MU and 2°
D12	Special procedure mode (TBI/TSET)	Annual	Output: same as regular beam; energy: same as regular beam; profile: same as regular beam

a Daily checks should be conducted for the energies used that day.

b Tolerance is the same as what was acceptable for TPS model evaluation at the time of commissioning.

**Table 3:** Mechanical tests

Item	Test	Frequency	Tolerance
M1	Localizing lasers	Daily	2 mm
		Monthly	1 mm
M2	Optical distance indicator	Daily	2 mm at isocenter
		Monthly	2 mm over clinical range
M3	Jaw position indicators	Daily	2 mm per jaw for single field
		Monthly	2 mm per jaw for clinical range of motion
M4	Light to radiation field coincidence	After Service	2 mm per jaw
M5	Leaf position accuracy	Monthly	1 mm
M6	Gantry/collimator angle indicators	Monthly	1°
M7	Physical graticule (port film graticule)	Monthly	2 mm
M8	Cross‐hair centering	Monthly	1 mm
M9	Treatment couch positions (absolute and relative)	Monthly	Abs: 2 mm and 1°; Rel: 1 mm over 10 cm and 0.5° over 3°
M10	Radiation isocentricity (MLC/jaw radiation isocenter with collimator, gantry and couch rotation)	Annual	2 mm diameter^a^
M11	Electron applicator collimator settings/physical inspection/interlocks	Annual	Same as acceptance/TPS
M12	Stereotactic accessories, lockouts, cone coding	Daily	Functional
		Annual	Functional
M13	Accessory latches/interface (all slots)	Annual	Functional

a For SRS‐SBRT applications, refer to the relevant AAPM Medical Physics Practice Guideline.

**Table 4:** Safety tests

Item	Test	Frequency	Tolerance
S1	Door interlock	Daily	Functional
S2	Door closing safety	After service	Functional
S3	Audio/visual monitors	Daily	Functional
S4	Beam‐on indicator	Daily	Functional
		Annual	Functional (all indicators)
S5	Anti‐collision test	Daily	Functional (single point for system function)
		Monthly	Functional (all collision interlocks)
S6	Safety procedures	Determined by QMP	Functional

**Table 5:** Wedge tests

Item	Test	Frequency	Tolerance
W1	Electronic wedge check	Daily	Internal: functional; collimator shaped wedges: 3%
		Monthly	2%
W2	Physical wedge placement accuracy	Monthly	1 mm
W3	Wedge profile for 60 degree electronic wedges, all energies	Annual	2% of TPS OAFs
W4	Wedge dose for collimator shaped wedges, all angles	Annual	2% of TPS dose

**Table 6:** Comprehensive review of machine settings

Item	Test	Frequency	Tolerance
C1	Comprehensive review of machine settings	Annual	Same as acceptance/expected

The tests are grouped together by class of test (e.g., dosimetry, mechanical, etc.) and clinical parameter tested. The recommended frequency and tolerance are listed with each test. Implementation notes on each test follow the tables. The recommended tests are also listed in more compact form in Appendix II. The appendix tables include an applicable question with each test to be completed by the QMP. This indicates that the QMP shall decide whether this test applies to their QA program based on the clinical use of the accelerator. Definitions:

Daily—this frequency implies that a specific test only needs to be done on the day the function is used.
Tolerance—all tolerances are listed as “within X% or within X mm” and they are listed to mean that the tolerance should be within ±X% or ±X mm of the standard or baseline. When a tolerance is listed as a percent change from a value (e.g., 2% of PDD), it indicates a relative change from the original value.

### D Dosimetry tests

#### D1 Photon and electron output constancy

Photon and electron beam output measurements had the highest RPN scores in the risk assessment. Therefore, it is recommended that output be measured daily, monthly, and annually.

- **Daily** and **monthly** output checks should be performed on all clinically used beams, and should fall within 3% and 2% of that system's baseline values, respectively. Daily checks may be restricted to the beams in clinical use for that day, at the discretion of the QMP. Readings outside these tolerances should be reported to the QMP to resolve the discrepancies and determine the appropriate course of action.
- **Annually**, output measurements must be performed in accordance with TG51 (or successor): in water with equipment calibrated by an accredited secondary standards laboratory within the previous 2 yr. Output for each beam must be within 1% of dose calculated via TG51 formalism. It is also recommended that the absolute calibration be externally validated.
- Once the beams are calibrated per TG51, secondary (monthly, if applicable) and tertiary (daily) measurement systems should then be irradiated to establish or confirm baseline output readings that are *tied to the primary calibration* (refer to section 8.A. of this report). The QMP may use a secondary measurement system (i.e., solid water based) for monthly output checks or use a water‐based system as done for annual calibration. The QMP must decide on the details of secondary and tertiary measurement systems; their fundamental attribute should be reproducibility.

The concept of acquiring or confirming annual baselines of secondary and tertiary measurement systems is described in detail of section 8.A of this report and shall also be applied to checks that follow: beam profile checks (D2) and beam energy checks (D3 and D4).

#### D2 Photon and electron beam profile constancy

- Most devices designed for **daily** output measurements also measure off‐axis constancy at one or more points in the radial and transverse planes. Results are displayed and saved as off‐axis factors (OAFs) or calculated as flatness and symmetry values. Facilities that possess such devices should monitor off‐axis constancy along with the daily output measurement.
- On a **monthly** basis, the QMP shall review the daily off‐axis measurements or measure beam profile shape with another device or method.
- **Annual** measurements of the beam profile must agree with off‐axis points in the TPS. Agreement of off‐axis points must be within 2% within the central 80% of beam as compared to the TPS data. The QMP should refer to MPPG5a for TPS commissioning recommendations.^21^ For geometries where the TPS model comparison to measured data is slightly greater than 2% due to modeling inaccuracies, the tolerance should be the same as what was achievable at the time of commissioning.
- A review by committee members of daily and annual profile measurements from different vendor accelerators and measurement devices revealed that an action level of 2% is a good compromise between detecting actual change in beam shape and false positives. The metrics used by most daily devices for beam profile constancy are flatness and symmetry. These are acceptable surrogates, although off‐axis point constancy is preferred.^22^, ^23^ Manufacturers of “daily check” devices are encouraged to provide direct readouts of off‐axis ratios, in addition to flatness/symmetry calculations.

#### D3 Electron beam energy

- Most daily measurement devices also measure electron energy constancy. On a **monthly** basis, the QMP shall review the daily energy measurements. The QMP may choose to take additional measurements with a second method, such as measurement at two depths in a phantom.
- **Annual** measurements of electron beam energy may be point measurements to verify I50 or R50 or measure a full ionization curve. If the I50/R50 measurement detects a change in energy, a full depth scan must be performed in water and if changes are found, the beam must be adjusted or recommissioned as needed.

#### D4 Photon beam energy

- Most daily measurement devices also measure photon energy constancy. On a **monthly** basis, the QMP shall review the daily energy measurements. The QMP may choose to take additional measurements with a second method, such as measurement at two depths in a phantom.
- **Annual** measurements of photon beam energy may be point measurements or a full depth dose curve in water. At a minimum, the QMP must verify the PDD_10X_ value used in TG51 calculations. Alternate measurements could be done to abide by any successive calibration protocol.
- Changes in OAFs have recently been shown to also be an indicator of photon energy change.^22^, ^23^ The QMP must choose the most appropriate method to monitor beam energy; however, the QMP must have full knowledge of the relationship between OAF changes or changes in PDD as it relates to changes in beam energy.

#### D5 Dynamic delivery control

Volumetric modulated arc therapy (VMAT) and sliding window techniques are types of dynamic deliveries routinely used that require the synchronization of the dose rate with other dynamic components of the machine. To produce a dynamic delivery, some combination of multileaf collimator (MLC) position, MLC leaf speed, dose rate, and gantry speed and position are varied throughout the treatment. Patient‐specific QA may not test the full range of these parameters, therefore, a **monthly** test of each of the dynamic control components used clinically is recommended. Tests have been designed to ensure the machine control of the individual dynamic components or to test them in combination by varying one dynamic control against another. Varian Medical Systems provides a series of tests for dynamic delivery along with the Digital Imaging and Communications in Medicine (DICOM) plans needed to execute them and spreadsheets to help with the analysis. In these tests, the gantry speed is varied against the dose rate control in one test and the MLC speed is varied against the dose rate control in another. Elekta provides similar tests at the time of acceptance. Or the user may design their own fields to test the different elements. With this type of delivery, a nonuniform dose delivery indicates a problem with the dynamic control. References and manufacturer recommendations indicate that the dynamic fields are able to deliver a dose within 3% of an open beam with the same dose objective.^24^, ^25^, ^26^ There are a wide range of available detectors, test designs, and interpretation software combinations that could be used. The QMP must decide what tests are important for their clinic and may wish to define a tighter tolerance depending on the sensitivity of each test/machine combination implemented clinically.

#### D6 Photon MU linearity (output constancy)

**Annually**, the QMP should test the clinical range of monitor units used for nonsegmented beams and the clinical range for MU/segment for segmented beams. Segmented fields (includes step‐and‐shoot and field‐in‐field) should be tested with the machine beam‐on/beam‐off control system that is used clinically for those types of deliveries. The dose per MU must be linear and agree to the dose per MU at the reference MU set (the MU used for calibration).

Static field MU linearity should be checked using the MU set on the accelerator down to the lowest clinically used MU setting. A review by committee members that work with all accelerator vendors suggests that a limit of 2% is achievable for open photon beams of 10 monitor units or greater.

Segmented field MU linearity should be checked by comparing dose from segmented fields to dose from the same open field using MU per segment down to the minimum allowed setting in the planning system. The ratio of dose from the static field with that from the segmented field must be within 2%.

#### D7 Electron MU linearity (output constancy)

**Annually**, the QMP should test the clinical range of MUs for electron beams. The dose per MU must be linear and agree to within 2% of the dose per MU at the reference MU set (the MU used for calibration).

#### D8 Photon output vs dose rate

**Annually**, all static and variable dose rates used clinically should be tested for output constancy. Dynamic dose rate control is tested in D5 and this may be sufficient for testing this parameter. The output must be within 2% of the nominal dose for all clinical dose rates.

#### D9 Photon and electron output vs gantry angle

**Annually**, photon and electron output vs gantry angle can be tested with an ion chamber in solid phantom at isocenter or with a gantry mounted diode/ion chamber array. A stable, reproducible setup is sometimes difficult to achieve for this test, therefore, an agreement of 2% of the output at the reference gantry angle (the one used during calibration – generally IEC gantry 0^o^) is appropriate.

#### D10 Photon and electron OAF vs gantry angle

As with output, OAF vs gantry angle can be a challenge to measure. A gantry mounted measurement system is very helpful, but not always available. **Annually**, the QMP should test clinically relevant angles for the facility. Single point measurements at some distance off‐axis (e.g., 10–15 cm) can be performed if an array is not available. Points off‐axis should agree with values at IEC gantry 0^o^ to within 2%.

#### D11 Arc mode (expected MU, degree)

This test is required **annually** if arc mode is used in a manner other than with dynamic deliveries (i.e., VMAT) such as static field arcs. This test must be performed for each energy and dose rate used clinically with arcs. The tolerance is 2% of the total MU of the arc and 2° over entire arc. If arcs are only used with VMAT deliveries and the QMP is doing VMAT patient specific QA and test D5, this test is not required.

#### D12 Special Procedure Mode (TBI/TSET)

**Annually**, critical clinical parameters used with any special procedures such as total body irradiation (TBI) and total skin electron therapy (TSET) should be tested. At a minimum, output, energy and OAFs should be verified for each special procedure mode at the clinical geometry with accessories in place. Testing accessories independently is not required if accessories are validated by using them in the measurement. Any special procedure mode not maintained for clinical use must be decommissioned.

### M Mechanical tests

#### M1 Localizing lasers

Many clinical facilities rely much less on lasers for patient setup than in years past due to daily image guided radiation therapy (IGRT) use. The QMP must determine the frequency and tolerances for laser tests. For example, the frequency and tolerance of testing for a treatment room that uses lasers as initial setup prior to IGRT should be less stringent than a room that sets up SRS patients for treatment with the lasers. Highly accurate SRS lasers should be verified by a more precise Winston–Lutz test as part of pretreatment patient QA thus we provide no specific tolerances for this use case. The tolerances should be specified by the QMP based on their uses.

#### M2 Optical distance indicator

**Daily** checks should include at least a check of the ODI at a single distance, typically 100 cm. **Monthly** checks should be done at multiple, clinically relevant distances, using whatever device the QMP deems appropriate (examples: mechanical front pointer, digital couch readouts). The tolerance should be 2 mm or the precision of the reading.

#### M3 Jaw position indicators

Individual jaw positions should be tested. Positions are typically checked **daily** with a single square field (10 × 10 or 20 × 20 cm^2^) using a jig or daily measurement device. Jaw positions should be tested against the readout at multiple settings across the clinical range of motion on a **monthly** basis. If jaws only operate in symmetric mode, then the pair of jaws should be checked. If jaws are used for beam‐splitting, jaw edge match should be within 1 mm at the central axis.

#### M4 Light to radiation field coincidence

The importance of the light field in photon treatments has diminished with the increased use of IGRT, although it is still necessary for setup of electron beams and some non‐IGRT beams. The QMP should decide on the frequency of this test based on their clinical practice. Some daily measurement devices are capable of measuring radiation field edge position which can be used to compare the jaw or MLC edge visible with the light field to the radiation field edge as part of the daily measurement.

At a minimum, the light to radiation field congruence should be verified **after service** to the mirror, field light bulb, or any work on the treatment head that may inadvertently affect the bulb or any component of the optical system.

#### M5 Leaf position accuracy

Positional accuracy of all leaves (and backup jaws, if applicable) should be checked **monthly**. It is the responsibility of the QMP to understand the MLC positioning system and decide which test is appropriate. The test should be performed at different gantry angles to detect any gravity‐induced positional errors. An acceptable test includes a Picket Fence‐type test.^27^, ^28^ Other tests that are tailored to the design of Elekta and Siemens MLC systems also exist (Hancock for Elekta and the Diamond jig system for Siemens). Leaves should move to prescribed positions to within 1 mm for clinically relevant positions.

#### M6 Gantry and collimator angle indicators

Test gantry and collimator angle readouts **monthly** at cardinal angles. If the imaging system uses a separate gantry encoder, it should be checked as well.

#### M7 Physical graticule

The port film graticule and digital graticules are used for different types of patient imaging systems. The QMP should test the type of graticule used clinically on each machine **monthly**. If a physical graticule is utilized it should be tested with a tolerance of 2 mm. If a digital graticule is utilized, the testing recommendations can be found in MPPG 2.a.^16^

#### M8 Cross‐hair centering

Cross‐hair centering is important for clinics that mark the central axis on the patient, use the ODI for patient setup or use the cross‐hair as a reference for isocenter during QA procedures. It is less critical for patient setup when using daily IGRT. The cross‐hair tray or mylar can be moved during service or cleaning, and thus require testing. Cross‐hair centering may be checked **monthly** by ensuring the diameter of the walkout is within 2 mm, thus ensuring the cross‐hair centering is within 1 mm.

#### M9 Treatment couch positions (absolute and relative)

Radiotherapy couches have between 4 and 6 degrees of freedom (DOFs). The absolute and relative tolerances for each of these DOFs will depend on the institution's workflow and procedures.

- Absolute Measurements: **Monthly**, test the absolute position of the table against the digital readout at a clinically relevant table position such as isocenter.
- Relative Measurements: **Monthly**, test the ability of the table to move a known amount to within 1 mm for translational moves and 0.5 degree for rotational moves over a clinical range. Test the table with any positioning systems used clinically to setup the patient (e.g., CBCT image guidance systems) and over all degrees of freedoms. For example, a phantom (with a corresponding reference position/image of the phantom) could be shifted to an offset position, imaged, shifted back to the reference position via the positioning system and re‐imagined or compared to reference marks to ensure that the table went to the correct location within the tolerance.

#### M10 Radiation isocentricity (MLC/jaw radiation isocenter with collimator, gantry, and couch rotation)

**Annually**, individual axis radiation isocenter tests can be done by creating spoke shot images. The runout on individual spoke shot images should circumscribe a circle that is ≤2 mm diameter. A Winston–Lutz type test that measures all three axes in a single test is preferred (the beam center should not deviate from the isocenter by more than a 1 mm radius (2 mm diameter) for any clinically used collimator/gantry/couch combination). The QMP should refer to MPPG9a for the frequency and tolerance of this test in a SRS/SBRT setting.^19^ If radiation isocenter tests indicate a problem, then the mechanical isocenter can be measured for each axis to help identify the problem.

#### M11 Electron applicator collimator settings/interlocks

Each electron cone that is used clinically should be tested for all available energies **annually**. The user should attach the cone and verify the machine code for the cone is read correctly and that the jaws drive to the correct positions. Each cone should be checked for physical integrity, as well as touch guards and interlocks including insert detections and coding.

#### M12 Stereotactic accessories, lockouts, cone coding

**Daily**, test the stereotactic accessories, couch lockouts, and cones (if applicable) used for patient treatments that day. **Annually**, verify the correct machine coding and jaw setting for all available circular cones if used.

#### M13 Accessory latches/interfaces (all slots)

**Annually**, verify that any accessory that mounts to the linac head latches properly and will not be dislodged or move in a way that will clinically affect the dose distribution position as the gantry rotates. This test is included to verify accessories that may not be included in M11, M12, or W2 (e.g., the block tray).

### S Safety tests

#### S1 Door interlock

The functionality of the door interlock should be checked **daily** to ensure that the radiation beam will terminate if the door is opened.

#### S2 Door closing safety

The QMP should ensure that the door is able to function in a safe manner when staff and patients enter and exit a treatment room. At a minimum, this test should be completed **after regular service** or any maintenance to the door. The QMP must consider testing the emergency opening options (e.g., battery backup, come‐along, etc.) for sliding doors or heavy swing doors. The QMP may determine an alternative frequency for this test based on the type of door and its opening design.

#### S3 Audio/visual monitors

The functionality of the audio and visual monitoring systems of the patient should be checked **daily**. At least one channel of audio and one channel of video monitoring are required for clinical use of the machine.

#### S4 Beam‐on indicators

The functionality of beam‐on indicators at the console and the door should be checked **daily**. All beam‐on indicators (inside and outside the vault) should be checked **annually**.

#### S5 Anti‐collision test

A single anti‐collision device should be checked **daily** for system function and each point may be rotated. All anti‐collision devices should be checked for functionality **monthly** if used clinically. These include laser guards and touch guards for imaging arms and the electronic portal imaging device (EPID). Electron cone touch guards are also checked annually in test M11.

#### S6 Safety procedures

The QMP should use knowledge and experience to determine a set of safety tests and the frequency that is necessary. These tests should be relative to clinical practice and technology used. The QMP may refer to manufacturer's guidelines and/or state regulations to determine which tests are appropriate; however, the QMP should decide on how these tests are implemented clinically.

### W Wedge tests

Definitions:

Physical Wedge—this term is used to describe a wedge that latches on to an accessory tray attached to the treatment head.
Internal Physical Wedge—this term is used to describe a wedge which is mounted and moves inside the treatment head (Universal wedge).
Collimator Shaped Wedge—this term is used to describe wedges formed by a moving collimator (Dynamic or Virtual wedge).
Electronic Wedge—this term is used to describe all internal physical wedges AND all collimator shaped wedges.

#### W1 Electronic wedge check

- The **daily** test for an internal physical wedge may be either a functional test that the wedge moves properly into the beam or an output measurement with the wedge in the beam. For collimator shaped wedges, it is recommended that the output be checked with the daily device to within 3% for the steepest wedge angle.
- **Monthly**, the QMP should review the daily wedge output results and investigate results that are consistently greater than 2% of expected. If daily output measurements are not made on the internal physical wedge, a monthly wedge factor should be measured with a tolerance of 2%.

#### W2 Physical wedge placement accuracy

On a **monthly** basis, verify physical wedge placement on the accessory tray and tray placement and latching onto the treatment head. A scribe mark on the wedge, tray, and tray slot can be used to verify repeatable positioning of the wedge. Test all wedges that are used clinically. Wedge placement should be consistent within 1 mm at the accessory tray.

#### W3 Wedge profile for 60‐degree electronic wedges, all energies

Wedge profiles should be measured **annually** for all clinically commissioned electronic wedge angles at a standard depth (typically 10 cm). A minimum of the 60‐degree wedge angle should be measured. Compare off‐axis points within the central 80% of the beam to TPS data used clinically. Agreement should be within 2% for all points.

#### W4 Wedge dose for collimator shaped wedges, all angles

The dose in wedged fields should be measured **annually** for all clinically commissioned collimator shaped wedge angles. The dose measurement can be done using absolute dose or wedge factors. Dose should agree to within 2% of TPS.

### C Comprehensive review of machine settings

#### C1 Comprehensive review of machine settings

The linac controller contains many definitions that describe clinical treatment parameters and machine configuration settings. Important examples include MLC leaf offset positions, collimator settings for electron applicators, etc. These definitions can have a large dosimetric impact if they are intentionally or inadvertently changed. Any machine configuration settings that were established at the time of acceptance and could impact the quality of the radiation beam if changed should be reviewed **annually**. It may be necessary to review these settings with the service engineer to obtain access to the information. Vendors are encouraged to provide tools to facilitate this review.

## 10 SUMMARY

This guideline will assist the QMP in developing a comprehensive QA program for linacs in the external beam radiation therapy setting. One deficiency of previous reports on linac QA testing is the lack of consideration for the clinical impact of failures of various tests performed. This committee sought to prioritize tests by their implication on quality and patient safety. Thus, the performance tests for linacs that are set forth in this guideline are derived from a combination of results from a risk analysis of currently recommended tests and the consensus of this committee. The tests presented in this guideline are intended to represent an acceptable level of QA standards that would ensure safe and high quality radiation treatments.

The QMP is ultimately responsible for implementing appropriate tests for their equipment taking into account the modality and complexity of treatments delivered, the diversity of patients and the level of image guidance involved. In the spirit of the report from AAPM Task Group 100^6^, individual institutions are encouraged to analyze the risks involved in their own clinical practice, use this guideline's recommendations as a minimum list of critical tests and determine which performance tests are relevant and prudent in their own radiotherapy clinics.

## ACKNOWLEDGMENTS

The Committee for Medical Physics Practice Guideline 8 of the Professional Council of the AAPM developed this guideline. Members: Koren Smith, MS, Chair; Peter Balter, PhD, FAAPM; John Duhon, MS; Gerald A. White Jr., MS, FAAPM; David L. Vassy Jr., MS, FAAPM, FACR; Robin A. Miller, MS, FAAPM; Christopher F. Serago, PhD, FAAMP, FACMP, FACR; Lynne A. Fairobent (AAPM Staff).

AAPM Subcommittee on Practice Guidelines—AAPM Committee responsible for sponsoring the draft through the process: Russell Tarver, MS, Chair; Jessica B. Clements, MS; Jennifer B. Smilowitz, PhD; Dustin Gress, MS; Per Halvorsen, MS, FAAPM, FACR; Arthur J. Olch, PhD, FAAPM; J. Anthony Seibert, PhD, FAAPM, FACR; Koren Smith, MS; Lynne A. Fairobent (AAPM Staff).

## CONFLICT OF INTEREST

There are no conflicts of interest.

## APPENDIX I

### RPN SCORES FOR LINEAR ACCELERATOR PERFORMANCE TESTS.

#### Table I

**Table:**

Rank order	Performance tests from TG142	Average RPN score^a^	Normalized RPN score
**Daily tests**			
1	X‐ray and electron output constancy	132	100
2	Stereotactic interlocks (lockout)	105	79
3	Laser localization	83	63
4	Collimator size indicator	70	53
5	Wedge: Morning check‐out run for one angle	55	42
6	Distance indicator (ODI) @ iso	41	31
7	Audiovisual monitor(s)	35	26
8	Door closing safety	33	25
9	Door interlock (beam off)	22	16
10	Radiation area monitor (if used)	12	9
11	Beam‐on indicator	11	9
**Weekly tests**			
1	MLC: Qualitative test (aka, “picket fence”)	101	100
**Monthly tests**			
1	X‐ray, electron, output constancy, backup monitor chamber constancy	143	100
2	Photon and electron beam profile constancy	120	84
3	MLC: leaf position accuracy (IMRT and Non‐IMRT)	113	79
4	Electron beam energy constancy	100	70
5	Localizing lasers	86	60
6	Wedge placement/compensator placement accuracy	74	52
7	Light/radiation field coincidence (asymmetric)	73	51
8	Jaw position Indicators (asymmetric)	72	50
9	MLC: travel speed (IMRT)	71	49
10	Wedge factor for all energies	69	48
11	Typical dose rate output constancy	68	48
12	Jaw position indicators (symmetric)	67	47
13	Accessory trays (i.e., Graticule or Dot Tray)	66	46
14	Light/radiation field coincidence (symmetric)	66	46
15	Digital graticule	63	44
16	Cross‐hair centering (walkout)	62	44
17	Gantry/collimator angle indicators	61	43
18	Distance check device for lasers compared to front pointer	61	43
19	Backup diaphragm settings (Elekta only)	60	42
20	Treatment couch position indicators	55	38
21	Laser guard‐interlock test	44	30
22	Latching of wedges, blocking tray	28	19
**Annual tests**			
1	X ray and electron output calibration (TG‐51)	183	100
2	TBI/TSET output calibration	114	62
3	X ray/electron symmetry change from baseline	113	62
4	X ray/electron flatness change from baseline	103	56
5	MLC: leaf position repeatability	94	51
6	Electron output constancy vs gantry angle	89	49
7	X‐ray output constancy vs gantry angle	87	47
8	X ray and electron off‐axis factor constancy vs gantry angle	83	46
9	Electron beam quality (R50)	83	45
10	MLC: moving window IMRT test	78	43
11	TBI/TSET PDD or TMR and OAF constancy	76	42
12	Physical wedge transmission factor constancy	74	41
13	MLC: segmental IMRT (step‐and‐shoot) test	74	40
14	X‐ray output constancy vs dose rate	73	40
15	Couch rotation isocenter	73	40
16	X‐ray beam quality (PDD10 or TMR 20/10)	72	40
17	X‐ray monitor unit linearity (output constancy)	71	39
18	Coincidence of radiation and mechanical isocenter	71	39
19	Wedge: off center ratio check (60 and intermediate angle)	67	37
20	Gantry rotation isocenter	63	35
21	Electron monitor unit linearity (output constancy)	63	34
22	Arc mode (expected MU, degree)	62	34
23	SRS arc rotation mode	59	32
24	Table top sag	59	32
25	MLC: coincidence of light field and x‐ray field (all energies)	58	32
26	MLC spoke shot	58	32
27	Stereotactic accessories, lockouts, etc.	57	31
28	TBI/TSET accessories	57	31
29	Collimator rotation isocenter	57	31
30	Output factors for electron applicators (spot check of one applicator/energy)	55	30
31	Spot check of field size dependent output factors for x ray (two or more FSs)	54	30
32	Table angle	50	27
33	MLC transmission (average of leaf and Interleaf): all energies	48	26
34	Safety: follow manufacturer's test procedures	38	21
35	Electron applicator interlocks	37	20
36	Table travel maximum range movement in all directions	34	19
37	TBI/TSET mode	24	13

a The standard deviation of the average value for O, S, and D was ≤ 3 for all tests scores.

## APPENDIX II

### MPPG 8.A RECOMMENDED PERFORMANCE TESTS

#### Table I

**Table:**

Item	Test	Frequency	Tolerance	Applicable to clinical practice?	QMP initials
**Dosimetry tests**					
D1	Photon and electron output constancy	Daily^a^ Monthly Annual	3% of baseline 2% of baseline 1% of TG51	Y/N Y/N Y/N	
D2	Photon and electron beam profile constancy	Daily^a^ Monthly Annual	2% 2% 2% of TPS OAFs^b^	Y/N Y/N Y/N	
D3	Electron beam energy	Monthly Annual	2 mm 2 mm	Y/N Y/N	
D4	Photon beam energy	Monthly Annual	1% of PDD/TPR (relative change in value) 1% of PDD/TPR at reference depth	Y/N Y/N	
D5	Dynamic delivery control	Monthly	3% of open field dose	Y/N	
D6	Photon MU linearity (output constancy)	Annual	2% >10 MU for open field; 2% for segmented field	Y/N	
D7	Electron MU linearity (output constancy)	Annual	2% for clinical range	Y/N	
D8	Photon output vs dose rate	Annual	2%	Y/N	
D9	Photon and electron output vs gantry angle	Annual	2% of IEC gantry 0° output	Y/N	
D10	Photon and electron OAF vs gantry angle	Annual	2% of OAFs at IEC gantry 0°	Y/N	
D11	Arc mode (expected MU, degree)	Annual	2% of MU and 2°	Y/N	
D12	Special procedure mode (TBI/TSET)	Annual	Output: same as regular beam; energy: same as regular beam; profile: same as regular beam	Y/N	
**Mechanical tests**					
M1	Localizing lasers	Daily Monthly	2 mm 1 mm	Y/N Y/N	
M2	Optical distance indicator	Daily Monthly	2 mm at isocenter 2 mm over clinical range	Y/N Y/N	
M3	Jaw position indicators	Daily Monthly	2 mm per jaw for single field 2 mm per jaw for clinical range of motion	Y/N Y/N	
M4	Light to radiation field coincidence	After Service	2 mm per jaw	Y/N	
M5	Leaf position accuracy	Monthly	1 mm	Y/N	
M6	Gantry/collimator angle indicators	Monthly	1°	Y/N	
M7	Physical graticule (port film graticule)	Monthly	2 mm	Y/N	
M8	Cross‐hair centering	Monthly	1 mm	Y/N	
M9	Treatment couch positions (absolute and relative)	Monthly	Abs: 2 mm and 1°; Rel: 1 mm over 10 cm and 0.5° over 3°	Y/N	
M10	Radiation isocentricity (MLC/jaw radiation isocenter with collimator, gantry, and couch rotation)	Annual	2 mm diameter^c^	Y/N	
M11	Electron applicator collimator settings/physical inspection/interlocks	Annual	Same as acceptance/TPS	Y/N	
M12	Stereotactic accessories, lockouts, cone coding	Daily Annual	Functional Functional	Y/NY/N	
M13	Accessory latches/interface (all slots)	Annual	Functional	Y/N	
**Safety tests**					
S1	Door interlock	Daily	Functional	Y/N	
S2	Door closing safety	After Service	Functional	Y/N	
S3	Audio/visual monitors	Daily	Functional	Y/N	
S4	Beam‐on indicator	Daily	Functional Functional (all indicators)	Y/NY/N	
		Annual			
S5	Anticollision test	Daily Monthly	Functional (single point for system function) Functional (all collision interlocks)	Y/N Y/N	
S6	Safety procedures	Determined by QMP	Functional	Y/N	
**Wedge tests**					
W1	Electronic wedge check	Daily Monthly	Internal: functional; collimator shaped wedges: 3% 2%	Y/N Y/N	
W2	Physical wedge placement accuracy	Monthly	1mm	Y/N	
W3	Wedge profile for 60 degree electronic wedges, all energies	Annual	2% of TPS OAFs	Y/N	
W4	Wedge dose for collimator shaped wedges, all angles	Annual	2% of TPS dose	Y/N	
**Comprehensive review of machine settings**					
C1	Comprehensive review of machine settings	Annual	Same as acceptance/expected	Y/N	

a Daily checks should be conducted for the energies used that day.

b Tolerance is the same as what was achievable for TPS model comparison to measured data as at the time of commissioning.

c For SRS‐SBRT applications, refer to the relevant AAPM Medical Physics Practice Guideline.

Signature of qualified medical physicist:

Date:
